# Left Atrial Myxoma Presenting As Multiple Embolic Strokes in a Young Female: A Case Report

**DOI:** 10.7759/cureus.98515

**Published:** 2025-12-05

**Authors:** Mandeep Singh, John Habashi, Tanvir Hassan, Anil Agarwal

**Affiliations:** 1 General Internal Medicine, Princess Alexandra Hospital, Harlow, GBR

**Keywords:** cardiac tumour, case report, cryptogenic stroke, embolic stroke, left atrial myxoma

## Abstract

Cardiac myxomas are rare benign tumors that can occasionally present with neurological complications due to embolic phenomena. We report a case of a 42-year-old woman who presented with sudden-onset slurred speech and transient right-sided weakness. Initial computed tomography (CT) of the brain and angiography were normal. Magnetic resonance imaging (MRI) revealed multiple small infarcts consistent with embolic strokes. Transthoracic echocardiography identified a large left atrial myxoma as the likely embolic source. The patient underwent successful surgical excision of the mass shortly after diagnosis. Neurologically, she showed progressive clinical improvement with no persistent focal deficits at postoperative follow-up, although she developed transient postoperative atrial fibrillation requiring anticoagulation. This case underscores the importance of early consideration of cardiac etiologies in young patients with multifocal embolic stroke and highlights the role of prompt echocardiographic evaluation and timely surgical management to prevent recurrent embolization.

## Introduction

Cardiac myxomas are the most common primary cardiac tumours, accounting for nearly 50% of all benign cardiac neoplasms, with approximately 75% arising from the left atrium, typically from the interatrial septum near the fossa ovalis [1]. Although histologically benign, their clinical significance stems from their potential to cause serious complications through embolic, obstructive, and constitutional mechanisms. The tumours are more frequently diagnosed in women, most commonly between the third and sixth decades of life [2]. Clinically, cardiac myxomas are notorious for their variable and often nonspecific presentation. Symptoms may mimic other cardiac or systemic conditions, usually delaying diagnosis. The classic triad of clinical manifestations includes obstructive features due to impaired intracardiac blood flow, embolic phenomena from tumor fragments or associated thrombi, and constitutional symptoms such as fever, malaise, or weight loss due to cytokine release [3]. Among these, embolic manifestations are particularly important because they may lead to cerebrovascular events, myocardial infarction, or peripheral arterial occlusion [4]. Approximately 30-40% of patients with atrial myxomas experience systemic embolization, and the central nervous system is the most frequent target [5].

In younger individuals without conventional vascular risk factors, embolic stroke can often be the first presentation of an underlying cardiac myxoma. Therefore, identifying cardiac sources of emboli in cases of cryptogenic stroke is of critical diagnostic and therapeutic importance. Echocardiography remains the primary diagnostic modality, with transthoracic echocardiography (TTE) detecting most lesions and transesophageal echocardiography (TEE) offering enhanced sensitivity for smaller or atypically located tumors [6]. Surgical excision is curative and usually results in excellent long-term outcomes when performed promptly [7].

We report the case of a 42-year-old woman without traditional cardiovascular risk factors who presented with multiple embolic strokes. Subsequent imaging revealed a large left atrial myxoma as the embolic source.

## Case presentation

A 42-year-old woman presented to the emergency department with a history of slurring of speech, consistent with a transient ischaemic attack (TIA), while attending a social event. The episode lasted for two hours, with spontaneous partial recovery. She reported burning sensations in both legs, had no correlating objective sensory deficit, denied headache, visual symptoms, limb weakness, or facial droop. There was no history of previous stroke, hypertension, diabetes, or smoking. Her only past medical history was Meniere’s disease.

Clinical findings

On examination, the patient was alert and oriented, with a Glasgow Coma Scale (GCS) score of E4 V5 M6, and an initial blood pressure of 160/96 mmHg and heart rate of 113 bpm. Neurological examination revealed intact cranial nerves, normal coordination, and no nystagmus or ataxia. Speech remained mildly slow, with no associated facial asymmetry, motor weakness, or objective sensory deficit. The transient elevation in blood pressure resolved spontaneously and was felt to be stress-related. Cardiovascular and respiratory examinations were unremarkable.

**Figure 1:**
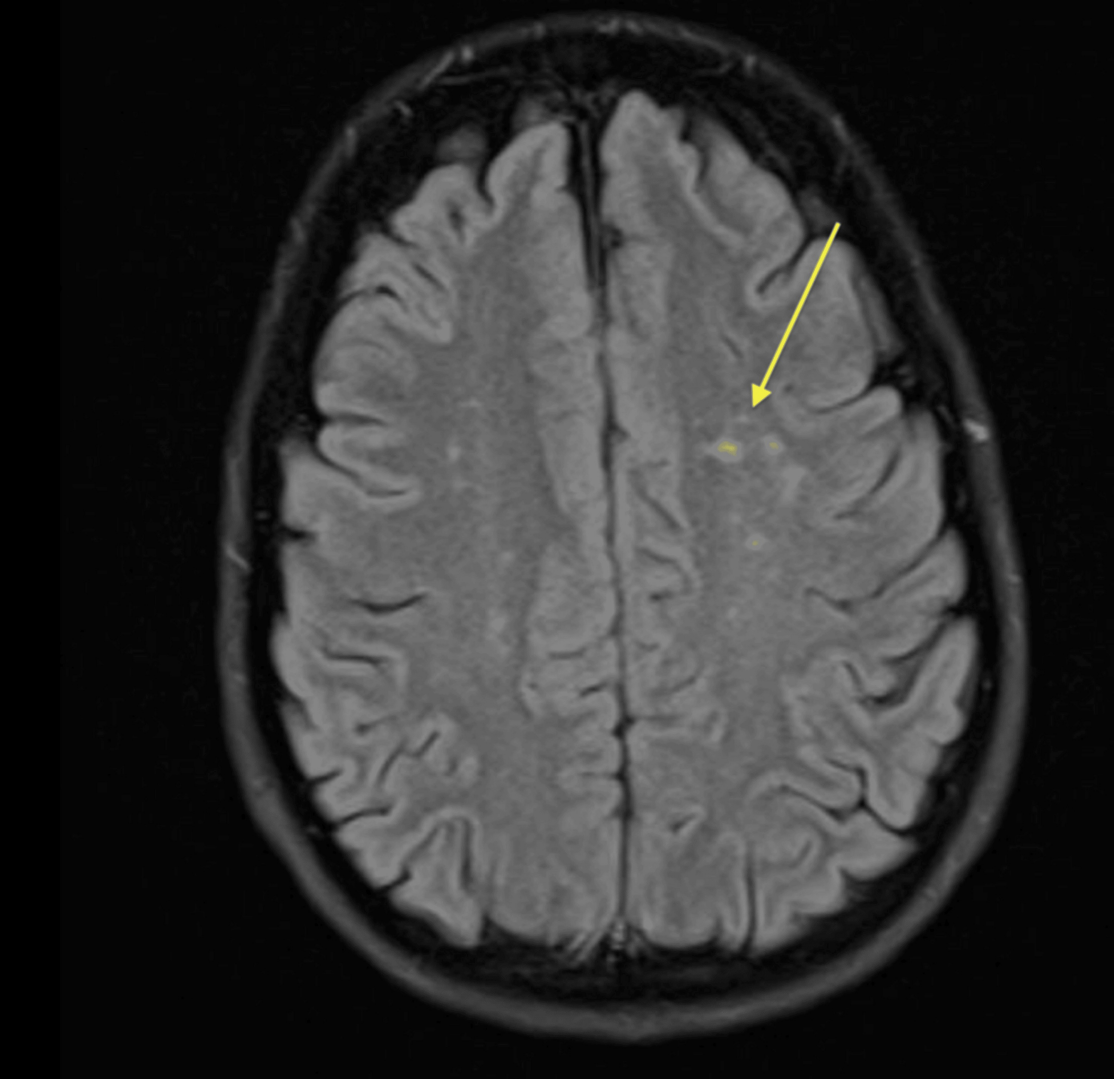
MRI head showing a small focus of restricted diffusion within the right thalamus, which may represent a recent infarct. A small subcortical focus of restricted diffusion is also seen in the left frontal lobe, with a further focus posteriorly in the left parietal lobe (yellow arrow).

A non-contrast CT of the head, CT angiogram of the aortic arch and carotids, and CT venogram were performed within the first 24 hours and demonstrated no acute intracranial hemorrhage, infarction, arterial stenosis, aneurysm, or venous sinus thrombosis. On day 2, MRI of the head showed multifocal areas of restricted diffusion in the right thalamus, left frontal, and left parietal lobes, consistent with recent embolic infarcts (Figure 1).

**Figure 2:**
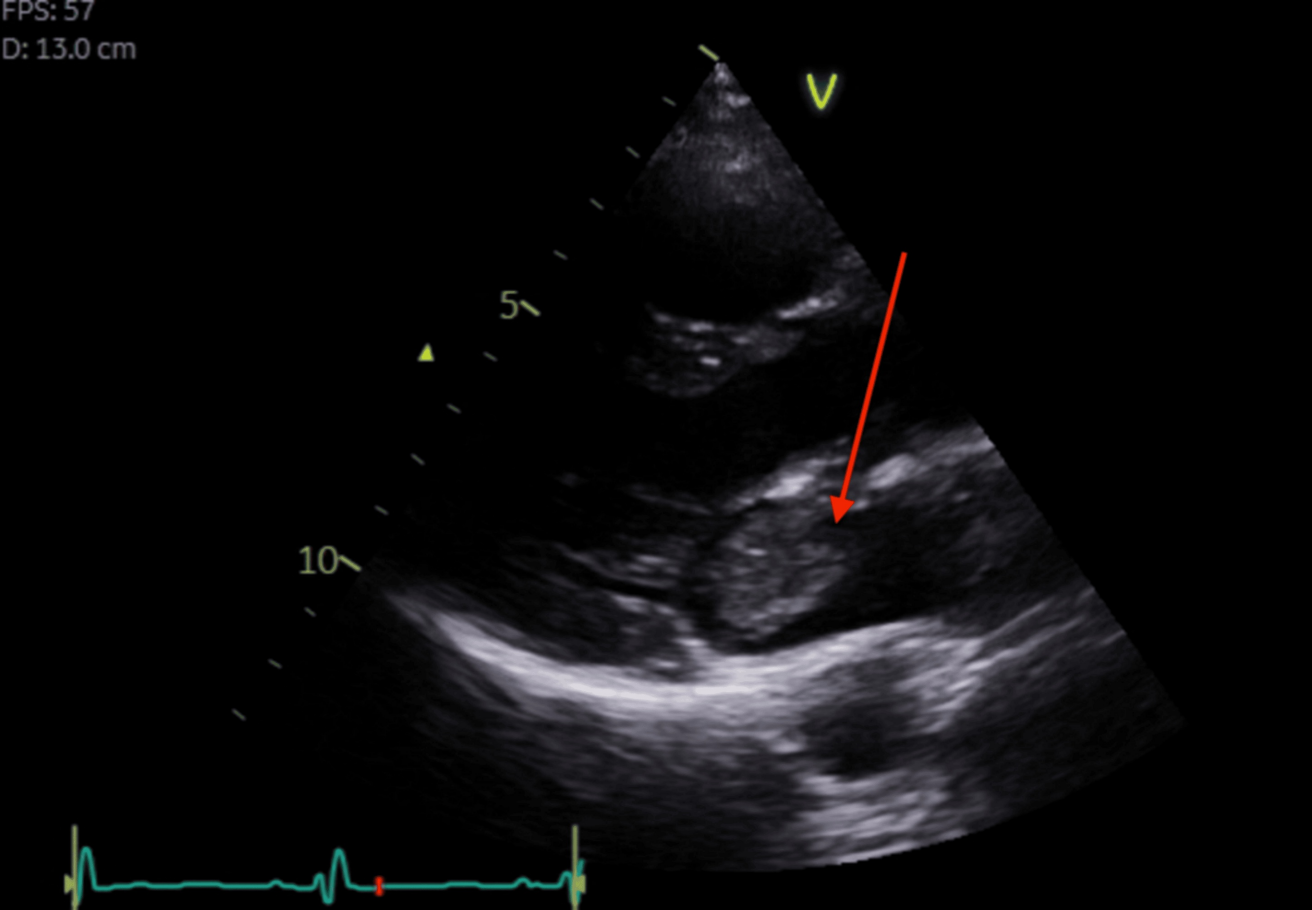
Transthoracic echocardiogram showing a mobile echogenic mass measuring 29 mm × 15 mm (red arrow), consistent with a left atrial myxoma.

**Figure 3:**
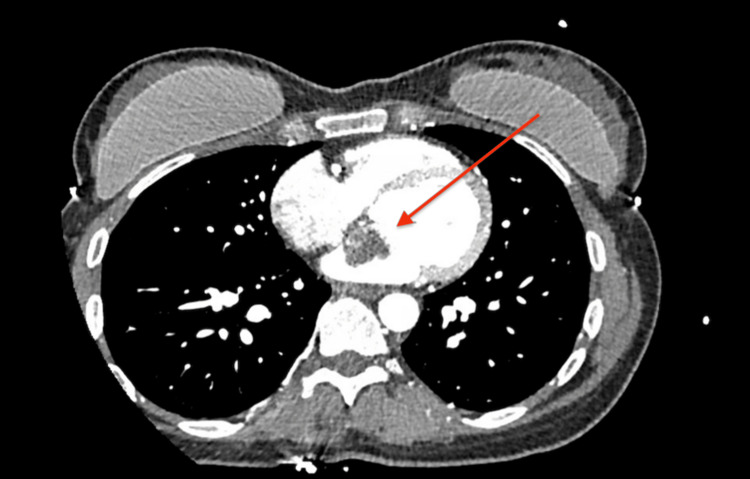
CT cardiac angiogram showing a 30 mm (red arrow) filling defect arising from the anterior wall of the left atrium, in keeping with the known history of myxoma.

Following cardiology input, TTE on day 4 revealed a mobile echogenic mass measuring 29 × 15 mm attached to the left atrial wall, consistent with atrial myxoma, as well as mild mitral and tricuspid regurgitation and preserved left ventricular ejection fraction of 60-65% (Figure 2). On day 5, a CT scan of the thorax, abdomen, and pelvis excluded embolic involvement of other organs. CT coronary angiography on day 7 identified a 30 mm filling defect in the anterior left atrium with no evidence of coronary artery disease (Figure 3). The patient subsequently underwent surgical excision of the atrial myxoma approximately two weeks after presentation, following multidisciplinary review.

Hospital course and management

The patient was initially treated for a possible transient ischemic attack with aspirin 300 mg daily. Following MRI confirmation of multiple embolic infarcts and echocardiographic identification of atrial myxoma, aspirin was discontinued, and therapeutic-dose enoxaparin was initiated based on stroke specialist recommendations.

The cardiology and cardiothoracic teams were engaged, and the case was discussed at a multidisciplinary meeting. After CT imaging excluded extracardiac emboli, the patient was transferred for surgical excision of the mass. Postoperatively, she developed transient atrial fibrillation that was successfully managed with bisoprolol and short-term rivaroxaban. Subsequent ECG and Holter monitoring confirmed sinus rhythm, and anticoagulation was discontinued. Histopathological evaluation confirmed the diagnosis of atrial myxoma.

## Discussion

This case demonstrates an embolic stroke attributable to atrial myxoma in a young adult without conventional cerebrovascular risk factors. Although stroke in young adults is less common than in older populations, it accounts for approximately 10-14% of all strokes and warrants careful evaluation for atypical or non-atherosclerotic causes [8].

In this case, a young patient presented with signs and symptoms of TIA and was treated with surgical removal of the myxoma after findings of myxoma on imaging, and was also medically managed for underlying symptoms. There is a correlation between patients presenting with embolic events and cardiac myxoma with ischemic stroke at a younger age, and with the size of the tumour, as was found by Qiao et al. (2022) in a retrospective study [9]. In our case, the absence of traditional vascular risk factors, coupled with multiple scattered infarcts on MRI and a mobile left atrial mass, is entirely consistent with the known embolic phenotype of left atrial myxoma. For example, in the report by O’Rourke et al., a 48-year-old man presented with bilateral infarcts due to left atrial myxoma measuring ~4 × 2.5×2.5 cm [10].

The distinctive feature in this case was the tumour’s mobility, moderate size (29 × 15 mm), and its intermittent prolapse through the mitral valve, both of which are recognized risk factors for embolization. The patient’s multifocal, bilateral infarcts (thalamus, frontal, parietal lobes) point to a proximal cardiac source rather than single-territory disease.

The timeline from diagnosis to surgical management is clinically important due to the risk of recurrent embolization. In this case, MRI confirmation of embolic infarcts was obtained soon after admission, echocardiography identified the atrial myxoma later that same week, and surgical excision was performed approximately 10-12 days after diagnosis. This timeframe reflected multidisciplinary collaboration between neurology, cardiology, and cardiothoracic teams and accounted for perioperative safety considerations in the context of a recent cerebral embolic event.

Postoperatively, the patient developed transient atrial fibrillation, a recognized occurrence after intracardiac surgery. She was commenced on rivaroxaban 20 mg daily and bisoprolol for rate control, with anticoagulation intended for a limited period of approximately four to six months. Discontinuation was planned only if a sustained sinus rhythm was demonstrated on ambulatory monitoring. A seven-day cardiac tape performed several weeks after surgery showed sinus rhythm throughout, and a subsequent 24-hour Holter was arranged to reassess the requirement for ongoing anticoagulation.

## Conclusions

This case highlights the importance of considering cardiac sources of embolism, including atrial myxoma, in young or middle-aged patients presenting with embolic stroke, particularly in the absence of conventional vascular risk factors or when neuroimaging demonstrates multifocal infarcts. Echocardiography remains the first-line modality for intracardiac evaluation, with TEE offering enhanced sensitivity and MRI/CT providing additional anatomical detail when required.

Timely identification and surgical removal of atrial myxoma can prevent further embolic events and is associated with excellent postoperative outcomes. Multidisciplinary collaboration between neurology, cardiology, and cardiothoracic teams is essential for optimal diagnosis, risk stratification, and management. Continued follow-up with rhythm monitoring and clinical surveillance remains important to detect potential recurrence or postoperative arrhythmias.
